# Quantitative analysis of retained austenite in Nb added Fe-based alloy

**DOI:** 10.1186/s42649-022-00074-1

**Published:** 2022-06-18

**Authors:** Kwang Kyu Ko, Jin Ho Jang, Saurabh Tiwari, Hyo Ju Bae, Hyo Kyung Sung, Jung Gi Kim, Jae Bok Seol

**Affiliations:** 1 Department of Materials Engineering and Convergence Technology, Gyeongsang National University (GNU), Jinju, 52828 Republic of Korea; 2 Department of Materials Engineering and Convergence Technology, Center for K-metal, Gyeongsang National University (GNU), Jinju, 52828 South Korea; 3 Department of Metallurgical and Materials Engineering, Gyeongsang National University (GNU), Jinju, 52828 Republic of Korea

**Keywords:** Retained austenite, LePera etching, Nital etching, Optical microscopy

## Abstract

The use of Pipelines for long-distance transportation of crude oil, natural gas and similar applications is increasing and has pivotal importance in recent times. High specific strength plays a crucial role in improving transport efficiency through increased pressure and improved laying efficiency through reduced diameter and weight of line pipes. TRIP-based high-strength and high-ductility alloys comprise a mixture of ferrite, bainite, and retained austenite that provide excellent mechanical properties such as dimensional stability, fatigue strength, and impact toughness. This study performs microstructure analysis using both Nital etching and LePera etching methods. At the time of Nital etching, it is difficult to distinctly observe second phase. However, using LePera etching conditions it is possible to distinctly measure the M/A phase and ferrite matrix. The fraction measurement was done using OM and SEM images which give similar results for the average volume fraction of the phases. Although it is possible to distinguish the M/A phase from the SEM image of the sample subjected to LePera etching. However, using Nital etching is nearly impossible. Nital etching is good at specific phase analysis than LePera etching when using SEM images.

## Introduction

Recently, the importance of pipelines as a long-distance transportation means for crude oil, natural gas, and similar applications is emerging. Up to now, the American Petroleum Institute (API) standard X80 or less has been applied to long-distance transport trunk line pipes. However, to improve the transport efficiency by increasing that transportation pressure, along with the improvement of the laying efficiency by reducing the diameter and weight of the line pipes, the increase in strength is accelerating. In particular, the X120 grade line pipe, which exhibits a tensile strength of over 900 MPa and can withstand approximately twice the internal pressure of X65, can transport about twice as much gas as compared to a lower grade line pipe of the same size (Hashemi 2011; Yoo et al. 2011).

Compared to the conventional methods of increasing the pressure enduring ability of the line pipe by increasing the pipe wall thickness, the use of high-specific strength line pipe saves the cost of materials, transportation, and on-site welding work which leads to overall construction cost savings. These enhanced properties achieved due to unique microstructure which is mainly comprised of martensite/bainite mixture.

The austenite transformation structure is classified into ferrite, pearlite, bainite, and martensite (Chipman 1972; Dube et al. 1958). The martensite phase forms through diffusionless transformation when austenite phase is quenched at a very high speed. According to the carbon content, martensite can be classified into lath/plate martensite (Krauss 1999). When the austenite is rapidly cooled, the carbon saturation concentration is reduced which leads to the super-saturated of carbon in the alloy phase. Also, the deformed region has high strength due to the high dislocation density. Since martensite has low elongation compared to strength, toe balance the strength ductility combination it is used by adding alloying elements such as manganese (Mn) and chromium (Cr) or increasing the carbon content (Krauss 1999).

According to the existing literature, the alloy of high strength-high ductility combination was mainly developed using the TRIP phenomenon reported by Zackay et al. (Zackay et al. 1967). It has a ductile ferrite matrix which is strengthened by bainite which gives it excellent mechanical properties such as dimensional stability, fatigue strength, and impact toughness owing to the combination of uniform elongation generated by the transformation from austenite to martensite (Rigsbee and VanderArend 1979; Daniel n.d.; Nishiyama 1978). A small amount of retained austenite remains even in two-phase steels, which have been studied as high-strength-high-ductility steels since the 1970s, and it’s reported that the ductility could be improved through transformation (Hayami and Frukawa 1975). In the late 1980s, various studies have been conducted to improve ductility by increasing the retained austenite fraction in the structure of abnormally structured steels (Matsumura et al. 1987; Chen et al. 1989). The volume fraction, distribution of various phases, especially the transformation rate and stability of retained austenite are very important in the mechanical properties of TRIP steel (Rigsbee and VanderArend 1979; Nishiyama 1978).

As mechanical properties can be determined according to the fraction of retained austenite, observation of microstructure in steel is very important. The microstructure observation of steel is generally divided into Black (pearlite, martensite), White (ferrite), and Gray (martensite, bainite) phases using Nital etching. However, in steel it is difficult to accurately distinguish phases in the images due to problems of contrast, especially in intermediate colors such as gray. It is well reported that LePera etching is capable of distinct phase analysis compared to Nital etching. Because LePera etching divides the phases into Blue/Green (ferrite), Brown (bainite), and White (martensite) (De et al. 2003; Santofimia et al. 2010; Gong et al. 2019). According to the research result of Tsipouridis (Tsipouridis 2006), the cooling rate has a large influence on the martensite structure. In the case of a slow cooling rate, the inside of unrecrystallized martensite grains is transparent white while for the fast-cooling rate, the inside of the coarsened grains appeared as dark brown.

In previous studies, the comparative analysis of the etching method is mainly performed based on microstructure of alloy. In this study, we aim to analyze the martensite and retained austenite (M/RA) phase of Fe-Nb alloy specimens through LePera etching which does not appear distinctly when using Nital etching. Nano-indentation tests have been performed to compare each phase. We are reporting concise inclusive optical microscopy(OM), scanning electron microscopy(SEM), electron backscatter diffraction(EBSD), and nano-indentation analysis for phase fraction analysis of Nb added Fe-based alloy.

## Experimental

In this study, an experiment was conducted using Nb-added bainite steel (hereinafter 4Nb) according to the composition of Table 1, to observe the microstructure of API Steel. After the alloy was prepared, the specimen was reheated and maintained at 1150 ° C for 2 hours, followed by initial rolling at a rolling ratio of 56%. Thereafter, final rolling was performed at a rolling ratio of 43% at 860 °C. After completing the rolling process, the specimen was cooled in water to 607 °C at a cooling rate of 4.1 °C/s, and then air-cooled to room temperature to prepare a specimen.

**Table 1:** Chemical composition of the alloys (wt.%)

Alloy	C	Si	Mn	P	Al	Cr	Ni	Mo	Ti	Nb	B	N
2 Nb	0.059	0.146	1.55	0.011	0.03	0.1	0.197	0.101	0.021	0.021	0.002	0.003
4 Nb	0.062	0.14	1.55	0.012	0.03	0.1	0.202	0.104	0.021	0.04	0.002	0.003
10 Nb	0.06	0.144	1.56	0.01	0.03	0.1	0.201	0.103	0.021	0.103	0.0021	0.003

The cross-section including the rolling direction and thickness direction of the specimen was subjected to Marco polishing from #200 to #4000 using SiC paper, micro polishing using 3 μm and 1 μm diamond suspension. An X-ray diffraction test was performed using an X-ray diffraction analyzer (X-ray Diffraction, XRD, Bruker, D2 Phaser). The voltage and current of the generator were set to 30 kV and 10 mA. The step size is 0.03° and the scanning range was set to 20 and 80°. XRD diffraction measurements were carried out using Cu Kα radiation (1.54184 Å) at room temperature, and the specimen was prepared by mechanically grinding the upper surface of the plate.

For microstructure observation, a LePera solution, which appears in various colors in the etching solution is used to clearly distinguish phases (De et al. 2003; Gong et al. 2019). The LePera solution can be obtained from a 1:1 volume ratio of Reagent 1 and Reagent 2 as mentioned in Table 2. The condition of the etched surface by this solution depends on the amount of Reagent 1, 2. When using an OM, it is necessary to raise the ratio of Reagent 2 when most phases show blue and increase the ratio of Reagent 1 to make a solution when most phases show brown. The experiment was conducted based on a LePera solution prepared by mixing 100 ml of ethanol +5 g dry picric acid +1.5 g Na_2_S_2_O_5_ + 100 ml distilled H_2_0, also about 4.7% Nital solution (ethanol 150 ml + Nitric acid 7.5 ml) was used for comparison with the LePera solution. The LePera solution was processed by adjusting the ratio of the mixed solution according to OM Image, and the etching time was used based on the experimental results of LePera (LePera 1979) and Santofimia (Santofimia et al. 2008). Thereafter, microstructure analysis was performed using an OM (Olympus BX53M), SEM (JEOL, JSM-7610F), EBSD (JEOL, JSM-7610F) (Acceleration voltage 20 kV, step size 0.20 μm, scan time 20 minutes, databases are BCC, FCC and HCP). The max load and acquisition rate are 30.0 mN and 10.0 Hz for nano indentation test. The loading and unloading rate are set equal to 200 mN/min.

**Table 2:** Composition of LePera solutions

Reagent 1	Reagent 2
1 g Na_2_S_2_O_5_ 100 ml distilled H_2_O	4 g dry picric acid 100 ml ethanol

## Results and discussion

**Fig. 1:**
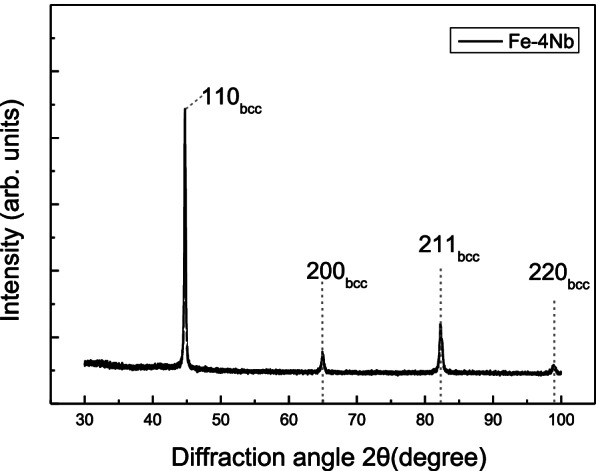
XRD analysis to determine the existing phase of 4Nb(wt.%) added Fe-based steel

Figure 1 shows the XRD pattern of the 4Nb specimen. The XRD pattern analysis confirms the BCC structure only, which refers that the residual austenite that can exist as a secondary phase after transformation has a very low phase fraciton which is beyond the measurement limit of XRD.

**Fig. 2:**
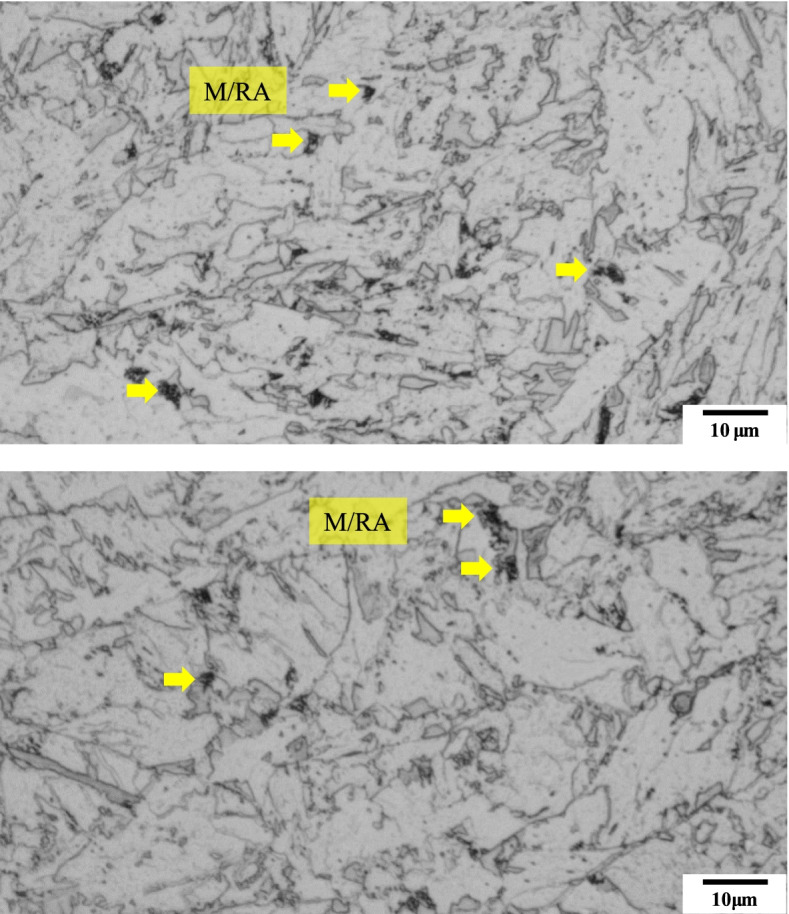
Optical micrographs subjected to Nital etching of 4Nb specimen. The marked phase is M/RA. But it can’t distinguish the ferrite phase and bainite phase due to similar color (white and gray)

Figure 2 shows microstructure image of the specimen after Nital etching. As it can be seen from the OM image, there is a limitation in clearly distinguishing the M/RAphases by Nital etching. To overcome the limitation of distinctly observing the phases the etching solution was changed to the LePera etching solution and the experiment is carried out.

**Fig. 3:**
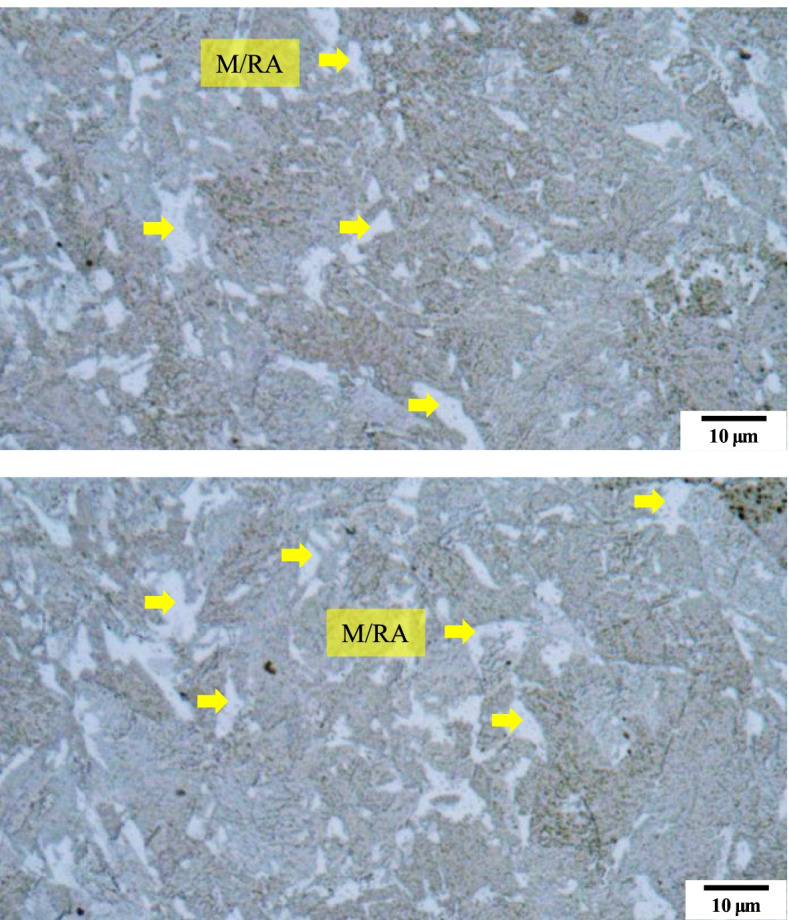
Optical micrographs subjected to Primary condition of LePera etching of 4Nb specimen. M/RA phases are observed but other phases have blur color

**Fig. 4:**
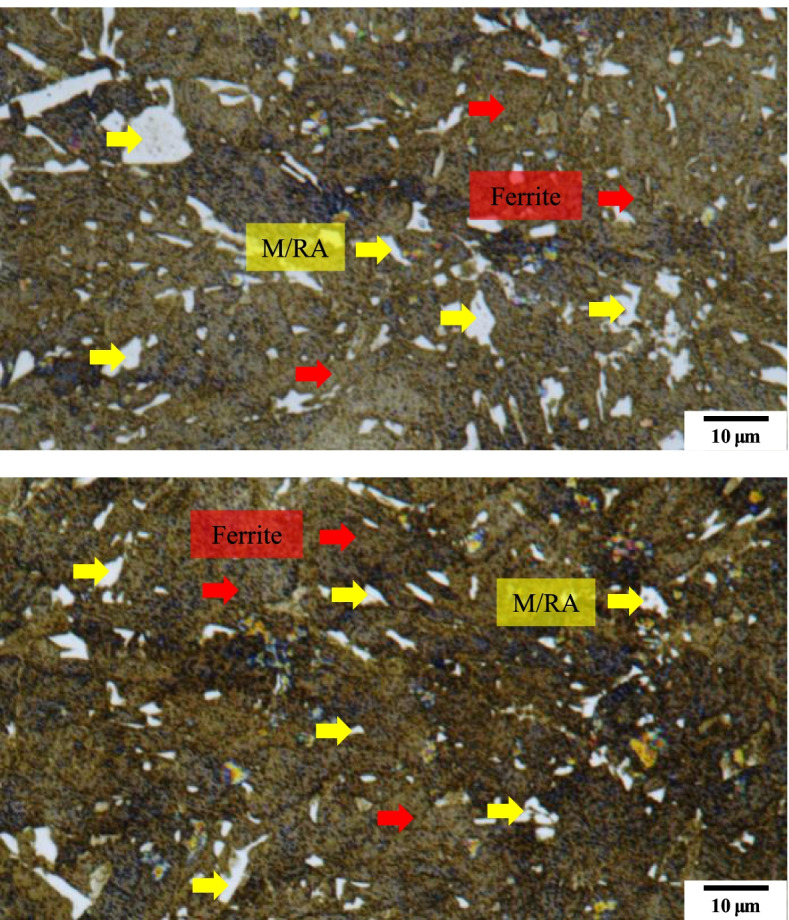
Optical micrographs subjected to Second condition of LePera etching of 4Nb specimen. In the same color as in the primary condition, M/RA is observed. Matrix(Ferrite) is dark brown, which means it is over-etched

**Fig. 5:**
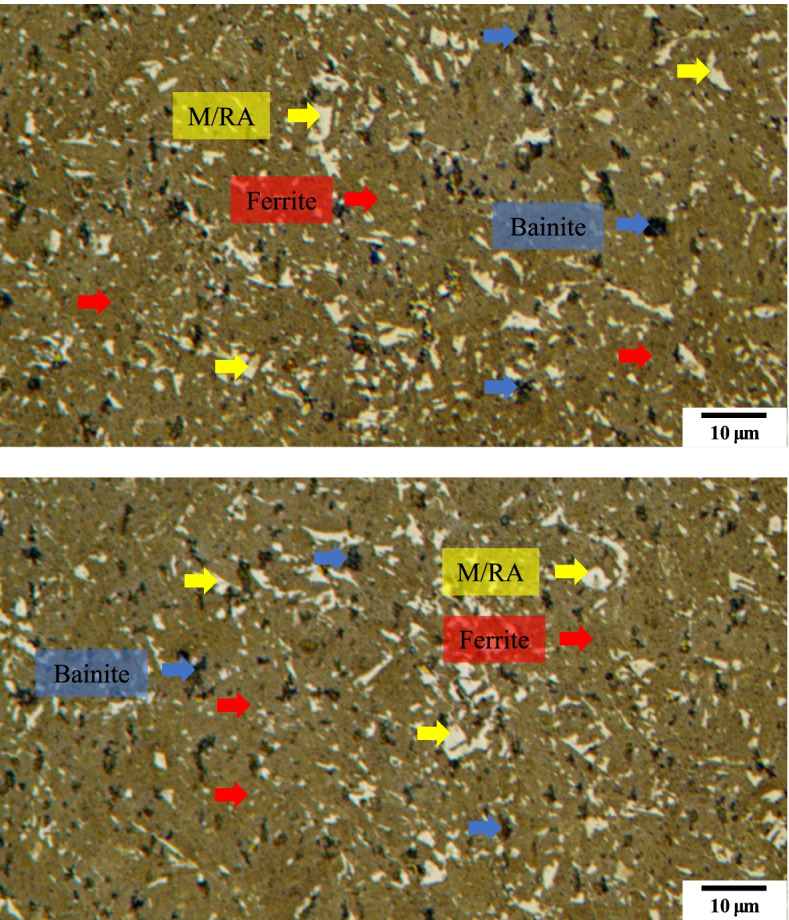
Optical micrographs subjected to Final etching conditions of LePera etching of 4Nb specimen. It can distinguish clearly the M/RA and matrix phase. Also, Bainite phases are observed in black

**Fig. 6:**
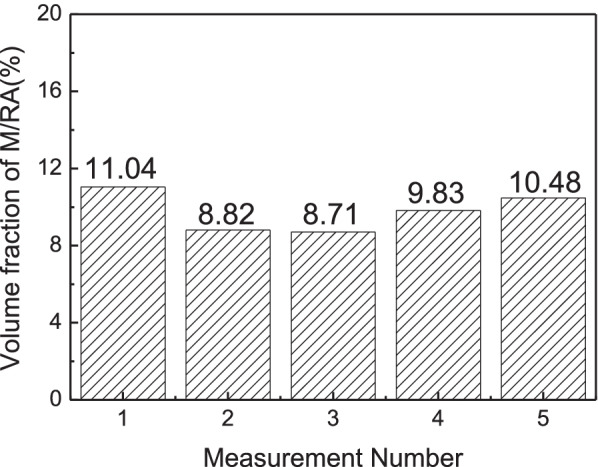
Results of M/RA volume fraction measured by optical micrographs subjected to Final LePera etching conditions of 4Nb specimen

Figure 3 shows the microstructure images as per the composition mentioned in Table 3. As the composition in Table 3 are the most widely known composition, M/RA phases are observed. Matrix is observed as yellow/blue color for ferrite phase whereas blur brown color for bainite. Etching conditions were changed as Table 4 so that the phase could be clearly distinguished. In Fig. 4, the M/RA phases appeared well, but the matrix was shown to be dark brown as a whole, which indicates over-etching. Figure 5 was obtained by experimenting again using Table 5 composition. It becomes accessible to clearly distinguish the M/RA phases. Also, the fraction of the M/RA phases could be measured with the ferrite microstructure images of the matrix. The fraction of retatined austenite and martensite was obtained using ImageJ software. Figure 6 summarizes the volume fraction of M/RA. It is measured to be 8–11% with the average of 9.77%.

**Table 3:** First composition of LePera solution

No.	Etchant	Content	Condition
1	Na_2_S_2_O_5_	1.0 g	30 s
2	Dry picric acid	4.0 g	
3	Ethanol (96%)	100 ml	
4	Distilled H_2_O	100 ml

**Table 4:** Second composition of LePera solution

No.	Etchant	Content	Condition
1	Na_2_S_2_O_5_	2.0 g	30 s
2	Dry picric acid	5.0 g	
3	Ethanol (96%)	100 ml	
4	Distilled H_2_O	100 ml

**Table 5:** Final composition of LePera solution

No.	Etchant	Content	Condition
1	Na_2_S_2_O_5_	3.0 g	30 s
2	Dry picric acid	7.0 g	
3	Ethanol (96%)	100 ml	
4	Distilled H_2_O	100 ml

**Fig. 7:**
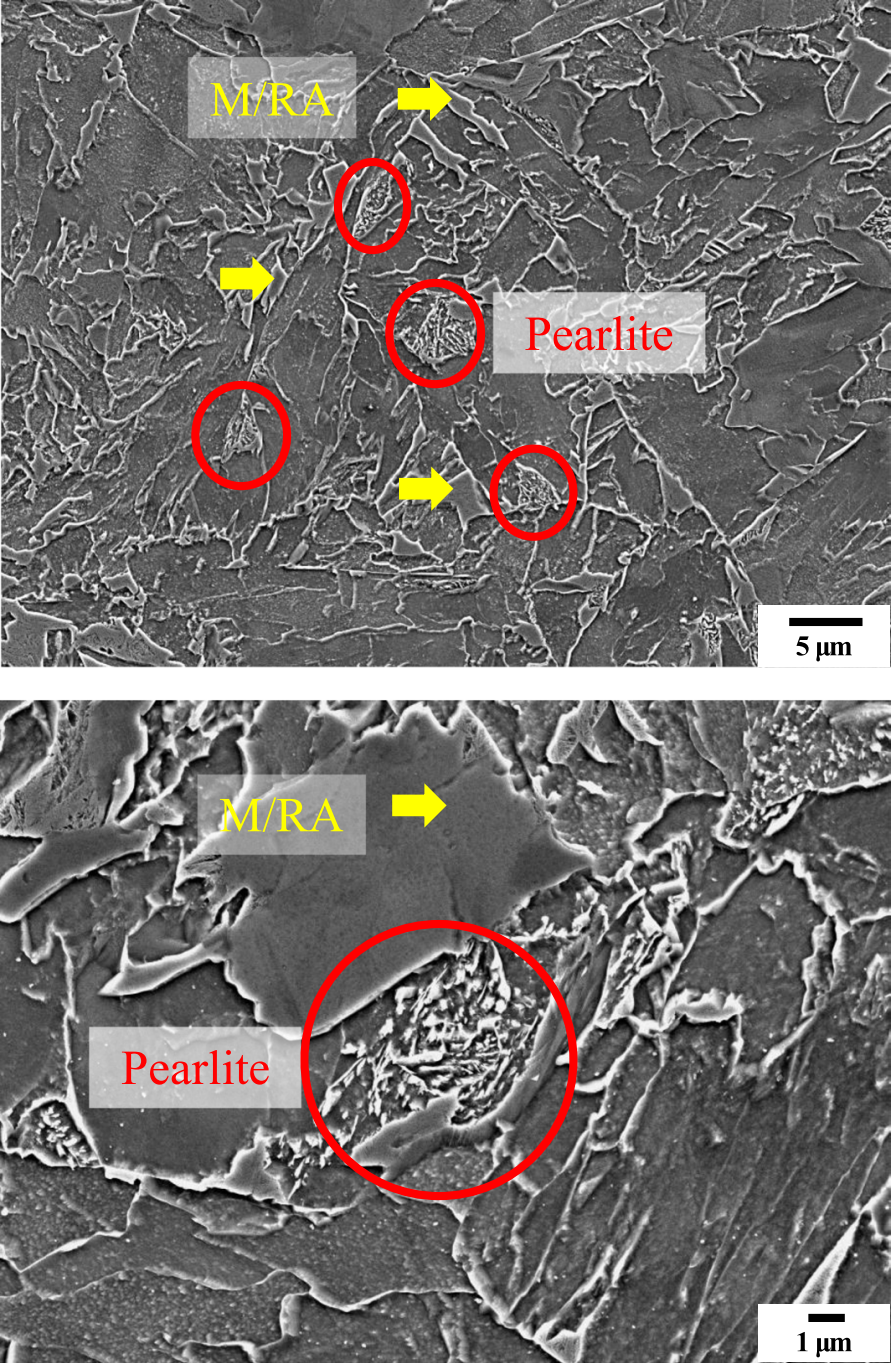
Scanning electron micrographs subjected to Nital etching of 4Nb specimen. M/RA phase and Pearlite phase are observed

**Fig. 8:**
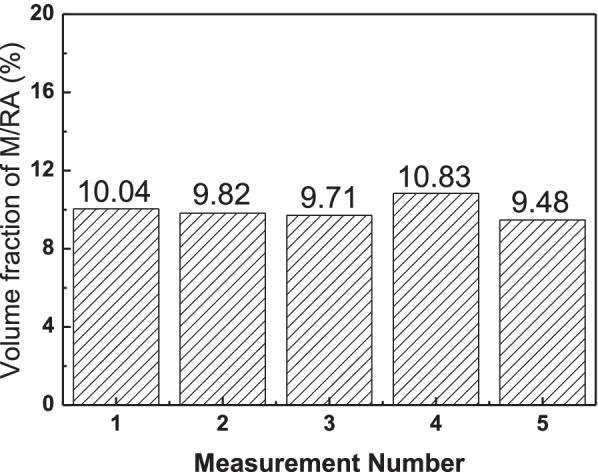
Results of M/RA volume fraction measured by scanning electron micrographs subjected to Final LePera etching conditions of 4Nb specimen

**Fig. 9:**
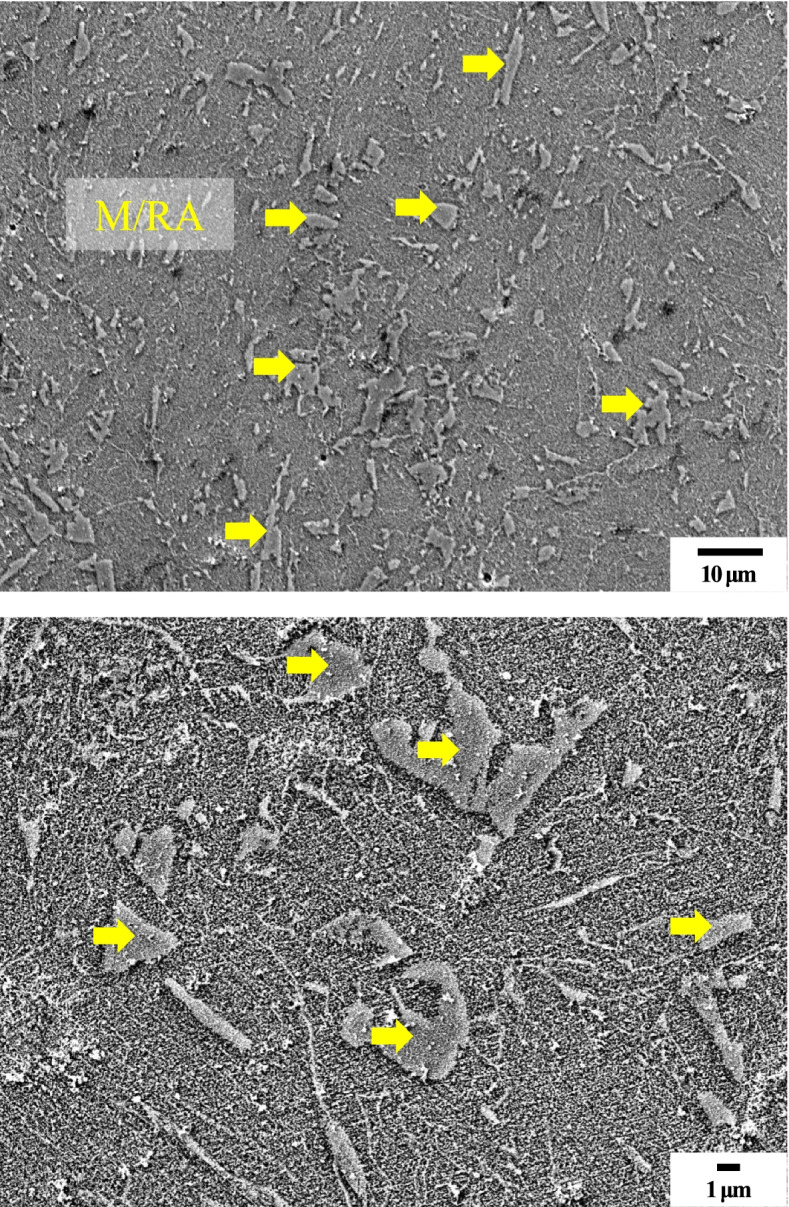
Scanning electron micrographs subjected to Final LePera etching conditions of 4Nb specimen. Although it was possible to distinguish the M/RA phase, it is difficult to distinguish other phases

Figure 7 shows the SEM images of specimen by Nital etching. Although there are difficulties in classifying the phases, the phase analysis is possible. Figure 8 shows the quantification of the M/RA volume fraction obtained from Fig. 9. Both SEM images and OM images are in agreement with a volume fraction of 9–11% and average 9.97%.

**Fig. 10:**
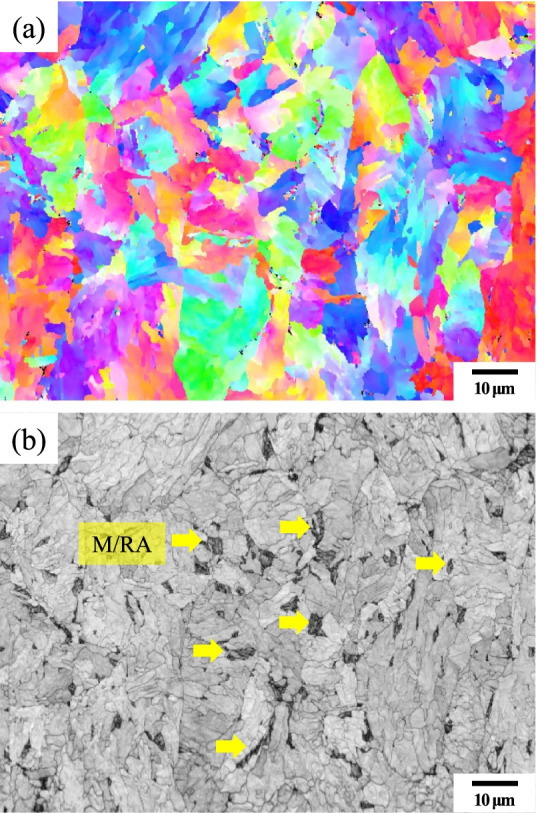
EBSD result subjected to Final LePera etching conditions of 4Nb specimen. (**a**) IPF map and (**b**) IQ map of 4Nb specimen

**Fig. 11:**
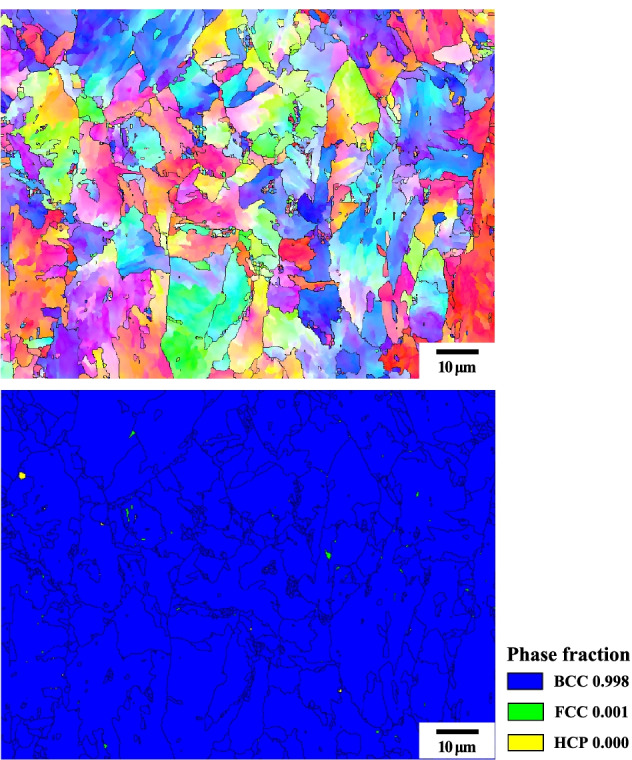
Phase map analysis subjected to Final LePera etching conditions of 4Nb specimen. Almost fully bcc phase was observed

Figure 10 is an IPF(Inverse pole figure) map of the 4Nb sample by LePera etching. The M/RA can be identified in this image. However, in the phase map, it is still observed as Fully BCC phase (Fig. 11). This supports that the M/RA phases should also be observed with OM in conjunction with XRD and EBSD.

**Fig. 12:**
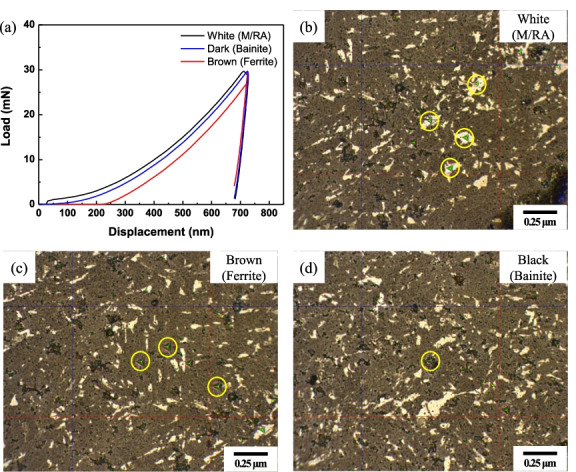
Results of nano indentation testing of 4Nb specimen subjected to Final LePera etching condition. (**a**) Load-Displacement curve, (**b**) White (M/RA). (**c**) Brown (Ferrite), and (**d**) Black (bainite) phases

Figure 12 is load-displacement curve with nano-indentation testing images. Through the difference in the Load-Displacement curve, it is possible to distinguish the phases according to the color, and to prove the distinction of the phases through LePera etching.

**Fig. 13:**
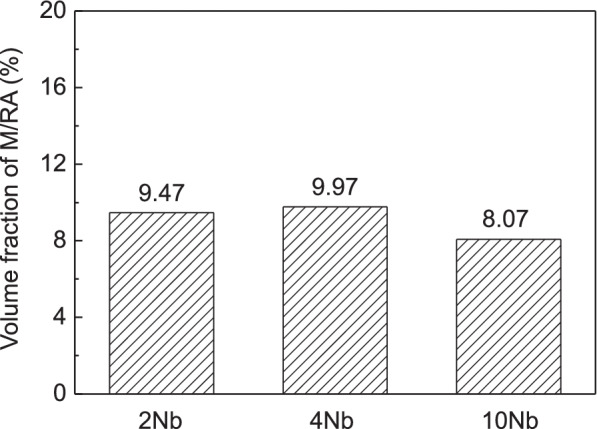
Results of M/RA volume fraction based on different Nb contents (wt.%)

**Fig. 14:**
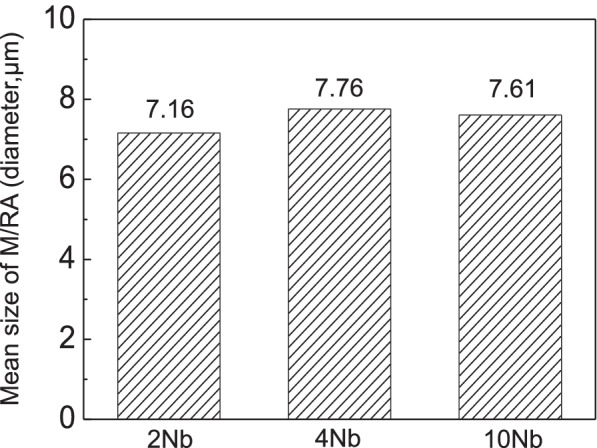
Results of M/RA mean size based on different Nb contents (wt.%)

Based on the above experimental method, the volume fraction and M/RA mean size is measured and compared for the 2Nb and 10Nb specimens in which only the Nb content has been changed to 0.02 and 0.10 wt%. Figure 13 shows the volume fraction of M/RA for 2Nb, 4Nb, and 10Nb specimens. 2Nb and 4Nb showed similar values, but for the 10Nb specimen, it decreased by 1.5 to 2% compared to the previous two specimens. Figure 14 shows the results of measuring the mean size of M/RA for three specimens. Unlike the M/RA volume fraction, 4Nb and 10Nb showed similar values, but 2Nb showed lower values than 4Nb and 10Nb. A further detailed study is needed on the correlation between the volume fraction and the mean size.

## Conclusions

M/RA phase transformation behavior is analyzed using a microstructure of Fe-4Nb alloy for API line pipe steel. For phase analysis at low temperature, the LePera etching method is used, and analysis is performed using OM and SEM images.

1. LePera etching was used to classify low-temperature phases (Bainite, Martensite), where Bainite was classified as Brown and Martensite as White. In particular, the martensite grain boundaries were uniformly distributed over the entire surface and mixed as white areas.
2. LePera etching conditions varies, depending on the steel composition. In this study, we found the most suitable etching condition of the specimen, Na_2_S_2_O_5_ 3.0 g, dry picric acid 7.0 g, and holding time 30s.
3. During Nital etching, the representative components constituting the microstructure, i.e., grain boundary ferrite, widmanstaten ferrite, acicular ferrite, etc., are clearly distinguishable and useful for quantitatively measuring each fraction, but the Martensite/Austenite (MA) phase is indistinguishable.
4. Similar results were obtained when measuring fractions in OM and SEM during LePera etching.
5. The M/RA phase could be observed on the OM image through LePera etching, and since it is difficult to obtain various information from the SEM image analysis, Nital etching is suitable for microstructure analysis.

## Data Availability

All relevant data have been included in the manuscript itself.
